# Intensified Surveillance and Insecticide-based Control of the Chagas Disease Vector *Triatoma infestans* in the Argentinean Chaco

**DOI:** 10.1371/journal.pntd.0002158

**Published:** 2013-04-11

**Authors:** Juan M. Gurevitz, María Sol Gaspe, Gustavo F. Enriquez, Yael M. Provecho, Uriel Kitron, Ricardo E. Gürtler

**Affiliations:** 1 Laboratory of Eco-Epidemiology, Department of Ecology, Genetics and Evolution, Universidad de Buenos Aires, Buenos Aires, Argentina; 2 Department of Environmental Studies, Emory University, Atlanta, Georgia, United States of America; Universidad Autónoma de Yucatán, Mexico

## Abstract

**Background:**

The elimination of *Triatoma infestans*, the main Chagas disease vector in the Gran Chaco region, remains elusive. We implemented an intensified control strategy based on full-coverage pyrethroid spraying, followed by frequent vector surveillance and immediate selective insecticide treatment of detected foci in a well-defined rural area in northeastern Argentina with moderate pyrethroid resistance. We assessed long-term impacts, and identified factors and procedures affecting spray effectiveness.

**Methods and Findings:**

After initial control interventions, timed-manual searches were performed by skilled personnel in 4,053 sites of 353–411 houses inspected every 4–7 months over a 35-month period. Residual insecticide spraying was less effective than expected throughout the three-year period, mainly because of the occurrence of moderate pyrethroid resistance and the limited effectiveness of selective treatment of infested sites only. After initial interventions, peridomestic infestation prevalence always exceeded domestic infestation, and timed-manual searches consistently outperformed householders' bug detection, except in domiciles. Most of the infestations occurred in houses infested at baseline, and were restricted to four main ecotopes. Houses with an early persistent infestation were spatially aggregated up to a distance of 2.5 km. An Akaike-based multi-model inference approach showed that new site-level infestations increased substantially with the local availability of appropriate refugia for triatomine bugs, and with proximity to the nearest site found infested at one or two preceding surveys.

**Conclusions and Significance:**

Current vector control procedures have limited effectiveness in the Gran Chaco. Selective insecticide sprays must include all sites within the infested house compound. The suppression of *T. infestans* in rural areas with moderate pyrethroid resistance requires increased efforts and appropriate management actions. In addition to careful, systematic insecticide applications, housing improvement and development policies that improve material conditions of rural villagers and reduce habitat suitability for bugs will contribute substantially to sustainable vector and disease control in the Gran Chaco.

## Author Summary

Vector-borne transmission of Chagas disease has not been effectively controlled in large parts of Latin America, particularly in the Gran Chaco ecoregion. To better understand the challenges in this region, we assessed the effectiveness of an intensified insecticide-based spraying strategy in suppressing the major vector *Triatoma infestans* from a well-defined rural area in northern Argentina. After an initial community-wide spraying, we intensively monitored infestation every 4–7 months for 35 months and applied insecticides selectively to the detected foci. In addition to the moderate levels of pyrethroid resistance reported in parallel, we found that selectively spraying only infested sites performed poorly. Prespraying bug abundance and the characteristics of infested sites before the initial interventions were reliable predictors of postspraying site infestation, including the type and use of the site and availability of refuges for the vector. We conclude that professional vector control based on residual insecticide spraying in an area with moderate pyrethroid resistance requires intense monitoring of house infestation, systematic insecticide applications and appropriate management actions. Operational, economic and political constraints to sustainable vector and disease elimination require complementary tools and approaches that favor changes in material conditions which reduce habitat suitability for the vector.

## Introduction

Field trials carried out in Brazil and Argentina in 1948 demonstrated the effectiveness of hexachlorocyclohexane for suppressing domestic infestations with *Triatoma infestans*, one of the major vectors of *Trypanosoma cruzi* [1], [2]. More than 60 years later, residual insecticide spraying continues to be virtually the only tactic applied to triatomine control. Chagas disease vector control programs typically have an initial ‘attack phase’ (in which full-coverage applications of insecticide are made) followed by a ‘surveillance phase’, in which vector detection surveys and selective insecticide sprays are implemented [3]. Decades of vector control actions and screening of blood donors dramatically reduced the numbers of infected people and population at risk; Brazil, Chile and Uruguay were declared free of blood-borne and vector-borne transmission of *T. cruzi* mediated by *T. infestans*, and the extent and intensity of infestations were substantially reduced in some sections of Argentina, Bolivia and Paraguay [4], [5].

The major obstacle to attain effective control of the major vectors of Chagas disease using residual insecticide spraying has been the reappearance of triatomine bugs and the difficulties in addressing this recurrent process. Reinfestation of human habitations and peridomestic structures after insecticide application has been documented for most of the main triatomine species [4], [6]–[8]. Sources of reinfestation for *T. infestans* have usually been associated with passive bug transport by people, active dispersal of bugs from residual or untreated foci, and more rarely and with less supporting evidence, from sylvatic foci [9]–[14]. For several species such as *Triatoma dimidiata*, *Triatoma brasiliensis* and *Rhodnius ecuadoriensis*, sylvatic foci represent the major source of bugs [15]–[18]. These species pose particular problems to vector control programs because they inhabit nearby vegetation where chemical control is hampered or infeasible.

Despite progress in vector control status, *T. infestans* and Chagas disease persist as a major public health problem in many rural and some periurban communities in the Southern Cone countries [11]. The initial goal of eliminating *T. infestans* set by the Southern Cone Initiative in 1991 has not been reached yet in the Gran Chaco region –a 1.1 million km^2^ semiarid plain covering large parts of northern and central Argentina, southeast Bolivia, and central and western Paraguay [19]. Several key factors converge in the Gran Chaco to maintain house infestation with *T. infestans*: suitable environmental conditions, hosts and habitats for bug development; poor living conditions; irregular vector control activities coupled with intrinsic operational difficulties (e.g., access through dirt roads, limited transportation); relatively few resources assigned to vulnerable populations with low political visibility and high disease burden; diminished effectiveness of pyrethroid insecticides because of environmental conditions [11], [20].

In the Argentinean dry Chaco, house reinfestation after insecticide spraying was mainly associated with the occurrence of residual foci in peridomestic structures [21]–[23]. Randomized field trials demonstrated that a double-dose application of pyrethroid insecticides produced a greater initial impact on peridomestic populations of *T. infestans* than standard doses and reduced house reinfestation rates in the dry Chaco [24], [25]. However, the understanding of house reinfestation dynamics still is very limited because only a few field trials assessed insecticide effectiveness in more than 100–200 houses during one year or more and monitored infestations once or twice per year [11], [26], [27]. These methodological details are relevant because the generation time of *T. infestans* may range from 4 to 6 months depending on temperature and resource availability [28], [29]. In addition, resistance to pyrethroid insecticides in *T. infestans* has been detected in northern Argentina and Bolivia [30]–[33]. The reasons for the lack of success of the regional elimination of *T. infestans* may be multiple and remain unclear.

As part of a multi-site research program on the eco-epidemiology and control of *T. infestans* in the Gran Chaco, we assessed the long-term impacts on house infestation and bug abundance of an intensified control strategy based on full-coverage pyrethroid spraying followed by frequent vector surveillance and immediate selective treatment of the detected foci in a well-defined rural area in northeastern Argentina. Before initial control interventions, a multi-model inference analysis showed that availability of appropriate refuges for *T. infestans*, use of cardboard as a building material, and household numbers of domestic hosts were strongly and positively associated with site-specific bug infestation and abundance, whereas reported insecticide use by householders was negatively related to infestation [33]. No sylvatic foci of *T. infestans* were detected [34]. Monitoring of house infestation during the first 12 months postspraying (MPS) revealed unexpected vector control failures associated with moderate levels of pyrethroid resistance [35]. By extending these observations with unprecedented levels of spatio-temporal detail and extent up to 35 MPS, we here focus on persistence of infestation at site or house level over time and space; assess the effects of selective treatments with a standard or double dose of pyrethroids, and conduct a multi-model inference analysis of factors putatively related to new infestations at site level detected at 12 MPS or subsequently. This investigation identifies several constraints operating on surveillance and subsequent insecticidal treatments that challenge Chagas disease vector suppression attempts in general.

## Materials and Methods

### Study area

Fieldwork was conducted in a well-defined rural section (450 km^2^) of the municipality of Pampa del Indio (25°55′S 56°58′W), Province of Chaco, Argentina (see map and photos in [33], doi:10.1371/journal.pntd.0001349.g001). The study initially encompassed all existing 353 houses and 37 public buildings in 13 neighboring rural villages. Newly-built houses during the three-year follow-up led to a final count of 411 different houses. The two main ethnic groups are Creole and Toba. Vector control activities in the area had historically been very sparse. The last community-wide insecticide spraying campaign conducted locally by vector control personnel was carried out in 1995; a few houses were treated by villagers or local hospital staff in 2006. Before community-wide residual spraying with pyrethroid insecticides in December 2007, the prevalence of infection with *T. cruzi* in bugs (27.4%), dogs (26%) and cats (29%) was indicative of active domestic and peridomestic transmission (M.V. Cardinal et al., unpublished results).

### Study design

A prospective cohort study was conducted between late 2007 and 2010. Surveys aiming at complete house coverage (i.e., a community-level census) were conducted at baseline and every 4–7 months during 35 months. A community-wide spraying with pyrethroid insecticides of all sites within each house compound was conducted immediately after the baseline survey [35]. Further interventions involved selective insecticide sprays of sites or house compounds infested during the follow-up period (Table 1). This study was approved by Institutional Review Board N° 00001678 (NIH approved) in Buenos Aires, Argentina.

**Table 1:** Insecticide applications performed in Pampa del Indio, 2007–2010.

MPS	Date	Spray type	Insecticide	Dose	No. of sites (houses) sprayed	Spray selection criteria
0	Nov–Dec/07	Community-wide	Deltamethrin	Standard	2,329 (348)^a^	All sites from all houses
4	Apr/08	None	None		0 (0)	None
8	Aug/08	Selective (site-level)	Deltamethrin	Standard or double	89 (52)	Only sites infested at 4 or 8 MPS and adjacent sites
12	Dec/08	Selective (site-level)	β-cypermethrin	Standard or double	27 (20)	Only sites infested at 12 MPS and adjacent sites
17	May/09	Selective (house-level)	β-cypermethrin	Double	347 (29)	All sites from houses with ≥1 site infested at 17 MPS
22	Oct/09	Selective (house-level)	Malathion	Standard	74 (8)	All sites from houses with ≥1 site infested at 22 MPS
28	Apr/10	Selective (house-level)	Malathion	Standard	87 (11)	All sites from houses with ≥1 site infested at 28 MPS
35	Oct/10	Selective (house-level)	Deltamethrin	Double	19 (3)	All sites from houses with ≥1 site infested at 35 MPS

MPS: months postspraying after the initial community-wide spray with pyrethroids.

a Including inhabited and uninhabited houses.

### Entomological surveys

Demographic and entomological surveys were conducted at baseline, during insecticide spraying, and at 4, 8, 12, 17, 22, 28 and 35 MPS. All existing houses were visited and its status recorded (inhabited, closed, abandoned, re-occupied, demolished, new). A sketch map of the spatial setting of all sites within each house compound was drawn, and each site was georeferenced and given an individual code in September or November–December 2007 (baseline, 0 MPS). The sketch map was updated during each visit. A house compound encompassed a domiciliary area with human habitations (sometimes in two separate buildings that counted as two domestic sites) and all sites within the peridomestic area (i.e., peridomicile) –usually a storeroom, a kitchen, an oven, one or more sites for chickens and other poultry (trees, coops, nests), one or more corrals, and a latrine. Each of these habitats characterized by some typical physical structure and use was considered an “ecotope”. A site (i.e., a patch) was any individual structure built and/or given a defined use by householders which might provide refuge for bugs.

All sites within each house were searched for triatomine bugs by timed manual collections (TMC) conducted by two skilled bug collectors using 0.2% tetramethrin (Espacial, Argentina) as a dislodging agent. Human habitations were inspected by one person for 20 min and each peridomestic site was searched by a second person for 15 min. In practice, each house compound averaged three peridomestic sites inspected and therefore the total search effort averaged one person-hour per house. In addition, most sites were inspected thoroughly before the stipulated time, and therefore search efforts were roughly similar across sites of different size. In several houses, bugs were also collected after the stipulated search time (after-manual collections), or by insecticide knock-down during insecticide applications (by the spray team) or a few days later (by householders) [35]. Local villagers were encouraged to capture bugs and hand them on to the research team during the subsequent visit. The collected triatomine bugs were transported to the field laboratory in plastic bags labeled with unique codes for house and bug collection site, identified taxonomically and counted according to species, stage and sex as described elsewhere [33].

### Insecticide application

The treatment criteria, insecticides and doses applied at different times are described in Table 1. The initial community-wide intervention sprayed all sites from 348 houses (including 325 inhabited and 23 vacant houses) with suspension concentrate (SC) deltamethrin (K-Othrin, Bayer, Argentina) at standard dose (25 mg/m^2^) applied by vector control personnel using backpack manual compression sprayers (Guarany, Brazil, and Hudson, Illinois) as described elsewhere [35]. Only four households refused insecticide spraying (not bug inspections) because they frequently sprayed themselves and their houses apparently were not infested, and another vacant house could not be accessed for treatment (Table S1). Selective sprays of all individual sites found infested with *T. infestans* at 4 or 8 MPS (including adjacent sites) were performed with deltamethrin upon completion of the 8 MPS survey. Likewise, sites found infested with *T. infestans* at 12 MPS (including adjacent sites within the same house compound and other sites that had not been sprayed at 8 MPS) were sprayed with SC β-cypermethrin (the only insecticide available to the vector control program at that time). To assess the impact of double-dose insecticide application on persistent infestations, standard (50 mg/m^2^) and double-dose (100 mg/m^2^) treatments with SC β-cypermethrin were assigned at random to infested peridomestic sites while a standard pyrethroid insecticide dose was applied in domiciles.

In view of the infestation levels recorded, from 17 MPS and thereafter the spray criterion was modified to full-spray coverage of infested house compounds (i.e., all sites within a house with 1 or more sites infested with *T. infestans* were treated with insecticide). Double-dose β-cypermethrin was used at 17 MPS. Field and laboratory-based evidence of local pyrethroid resistance [35] supported the application of a standard dose of malathion (1 g/m^2^) –the only effective alternative to pyrethroids available that was authorized by the corresponding federal agency at that time– to the few house compounds still infested with *T. infestans* at 22 and 28 MPS. At 22 MPS, one house was left unsprayed by mistake, and the owner of another house refused spraying; both houses were sprayed at 28 MPS.

### Data analysis

All data reported correspond only to inhabited houses unless otherwise noted; no public building and only two uninhabited houses were ever found infested with *T. infestans* in the study area. The prevalence of infestation and colonization by *T. infestans* was computed either for sites or house compounds. Infestation was defined by the catch of at least one live *T. infestans* nymph or adult, and colonization by the catch of at least one *T. infestans* nymph. Persistent infestation of a site (or house) at time *t* was defined as the occurrence of infestation in a given site (or house) both at time *t*−1 and *t*. Estimates of infestation prevalence were based on the combined results of TMC and knock-down bug collections. Householders' bug collections were only considered when provided with precise information on date and site of capture; these and other data were used to distinguish between occasional invasions and established infestations. Bug abundance was computed as the number of live *T. infestans* collected in a specific site per 20 (domiciles) or 15 (peridomestic sites) person-minutes of search effort by TMC. As a measure of insecticide spraying effectiveness at site- or house-level, the percentage of sites (or houses) infested and sprayed at time *t* that were again found infested at time *t*+1 (i.e., apparently were persistently infested) was calculated for each selective spray round.

Data on reported insecticide use, ecotope, building materials, refuge availability, household numbers of people and domestic animals, and host resting places were collected in every survey starting on 4 MPS. The corresponding data for 0 MPS were extrapolated from the 4 MPS survey as explained elsewhere [35]. Demographic data for the 35 MPS survey were taken from the preceding survey at 28 MPS; although this procedure may introduce some inaccuracies, these should be trivial because only three sites were found infested at 35 MPS.

The association between new site-level infestations detected at 12 MPS or subsequently and refuge availability, reported insecticide use by householders, and distance to the closest infested site at *t*−1 and *t*−2 surveys was evaluated by means of multiple logistic regression analysis. Apparently new infestations were only considered from 12 MPS onwards; houses found infested at 4 or 8 MPS were excluded from this analysis because they had high chances of being locally persistent foci –at site or house level– after initial interventions. Thus, for this particular analysis, an infestation occurring at time *t* was considered new (i.e., not persistent) if it was found at 12 MPS (or subsequently) in a house considered uninfested at 4 and 8 MPS (or at *t* – 1). A multi-model inference approach based on Akaike's Information Criterion (AIC) was used to assess the relative importance (RI) of each variable [36] as detailed elsewhere [33]. The maximum value RI can take is 1, representing maximum relative importance, whereas RI = 0 represents no importance at all relative to the set of variables considered. Parameter estimates for each predictor variable were based on averaging the parameter value in each model including the predictor weighted by the Akaike weight of the respective model. Analyses and calculations were performed in R 2.7.0 [37].

The spatial distribution of houses with persistent infestations at 4 MPS was evaluated with respect to house infestation at 0 MPS (i.e., most bug colonies with late stages found at 4 MPS were very unlikely to have established after the initial insecticide spray because of the long generation time of *T. infestans* ranging from 4 to 6 months). The null model was built maintaining the pattern of infestation at 0 MPS (pattern 1) fixed, and randomizing the status of infestation of houses at 4 MPS (pattern 2) among all existing houses. The *O*-ring statistic *O*_12_(*r*) [38] was used to evaluate if the number of points of the randomized pattern 2 within a ring of radius *r* and a given width, centered at each point of the fixed pattern 1, corresponded on average to a random process (i.e., a homogeneous Poisson process; *O*_12_(*r*) = 1); aggregation of 2 relative to 1 (*O*_12_(*r*)>1), or regularity of 2 with respect to 1 (*O*_12_(*r*)<1). This procedure was implemented in Programita [39]. The grid size for analysis was 100 m; ring width, 400 m; maximum radius, 5 km; 999 simulations were performed, and the upper and lower 25^th^ simulations were used as a 95% confidence envelope. A goodness-of-fit test was used to evaluate the overall fit of the observed pattern to the expected distribution [39].

Using the prospective data available, we assessed the hypothetical effects of adopting alternative insecticide spray criteria to the ones actually adopted (Table 1). The alternative criteria were either to spray all the sites within a house compound with one or more infested sites (i.e., the criterion applied originally at 17 MPS and thereafter), or to spray all sites within a given radius from the sites found infested. For each of the first four selective sprays rounds, we identified the sites that would have been sprayed at survey *t* if an alternative criterion had been applied; e.g., for the first criterion, by identifying all the sites within a house compound with an infested site at a given survey. For these identified sites, we searched for sites that were infested at survey *t*+1 and recorded the outcome at survey *t*+2, had these sites been sprayed at survey *t*+1. The outcomes at *t*+2 were then taken as the hypothetical outcomes for *t*+1 under the alternative criterion for the identified sites; for other sites, the observed infestation for *t*+1 was considered. Taken together, these outcomes represented the hypothetical infestation status under the alternative spray criteria for each survey. Distances between 0.1 and 5 km at 0.05 km increases were considered as hypothetical spray radii. As these criteria imply spraying more sites than those that were actually sprayed with insecticides, infestation would decrease solely because of the fact of treating more sites and not because nearby sites were treated. This procedure was used to calculate confidence envelopes for the hypothetical infestations. For each distance considered, as many sites as those that would be sprayed were randomly selected and the same calculations as with the actual sample were performed. This procedure was repeated 1,000 times for each distance and survey, and the upper and lower 25^th^ values were taken as the 95% confidence envelope. Calculations were performed in Matlab 7.3.0 [40].

## Results

### Study population

All of the 411 houses enumerated during the period September 2007–October 2010 were included in this study, although not all of them occurred at the same time point. Few (4–6%) houses were vacated between consecutive surveys, whereas newly-built or re-occupied houses represented 4–5% of the total number of inhabited houses at each survey. Very few households refused searches for bugs through the follow-up (Table S1). The main reason for lost-to-house inspection was that residents were repeatedly absent and access to closed premises through neighbors could not be arranged. A total of 4,053 sites was inspected for infestation at least once.

### Insecticidal effects

**Figure 1:**
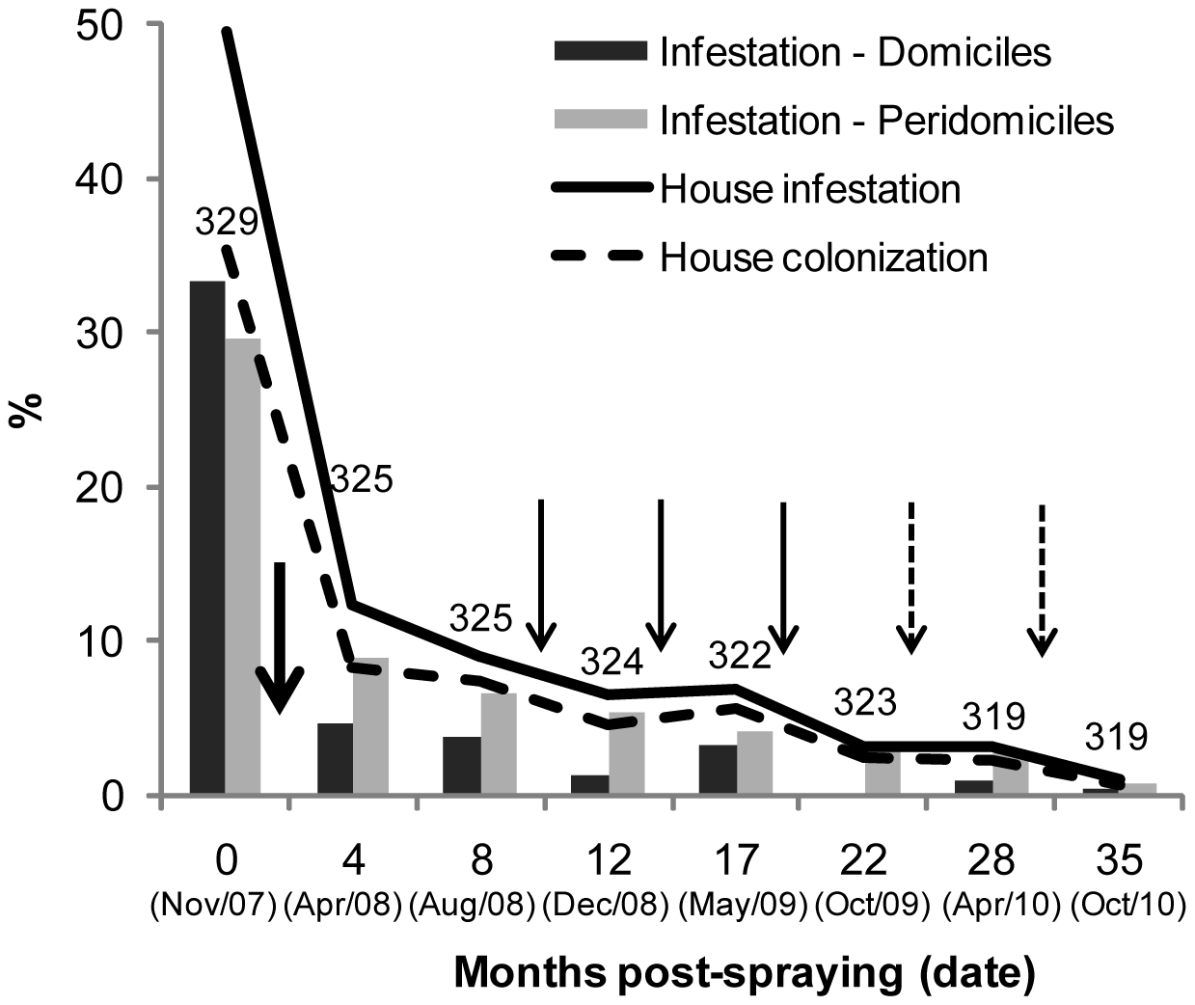
Infestation and colonization with *T. infestans* and interventions performed during the 35-month follow-up. Pampa del Indio, 2007–2010. Numbers above bars indicate number of houses inspected for infestation. The bold arrow indicates the initial community-wide insecticide spraying; thin full arrows indicate selective sprays with pyrethroid insecticides (either infested sites or infested house compounds), and thin dashed arrows indicate selective sprays with malathion of infested house compounds.

The initial community-wide insecticide spraying reduced the overall prevalence of house-level infestation with *T. infestans* from 49.5% before interventions to 12.3% and 8.9% at 4 and 8 MPS, respectively (Figure 1). After each of the first two selective treatment rounds conducted at 8 or 12 MPS, overall house infestation remained at 6.5–7% at 12 or 17 MPS. After the third selective treatment at 17 MPS, when all sites in any infested house were sprayed with a double-dose of pyrethroids (Table 1), overall house infestation fell to 3% at 22 or 28 MPS, and below 1% after selective treatments with malathion.

**Figure 2:**
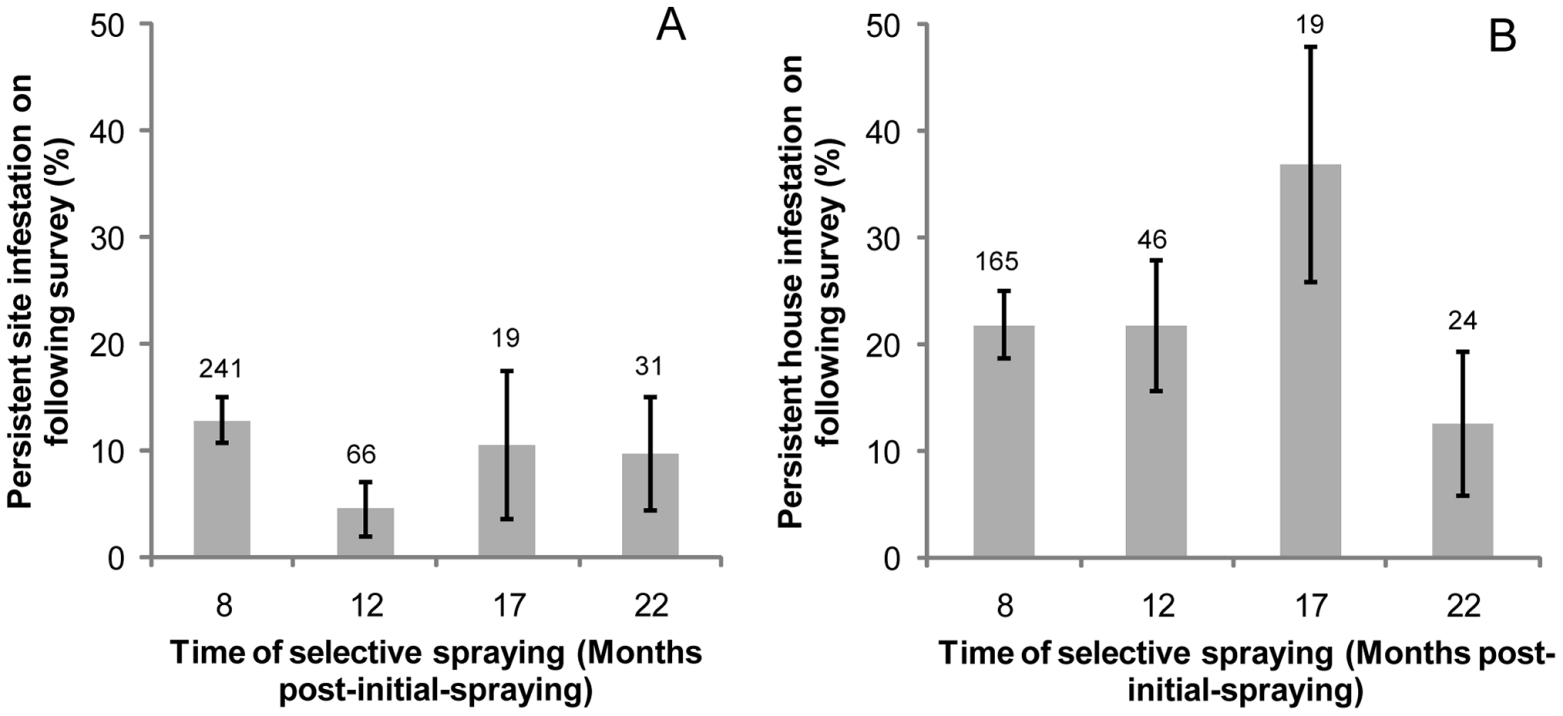
Persistent infestations with *T. infestans* after each selective spray round. Pampa del Indio, 2007–2010. A: Site-level persistence of infestation. B: House-level persistence of infestation. Whiskers represent ±1 standard error; numbers above bars are number of observation units (sites or houses) infested, sprayed, still existing and re-inspected at the subsequent survey after the selective spray.

We assessed the effectiveness of selective pyrethroid sprays in suppressing site-specific infestations. TMC searches conducted 4–6 months after each of the first four pyrethroid spray rounds revealed that 5–13% of the treated sites were persistently infested between successive surveys (Figure 2A). No significant differences in effectiveness were detected between selective spray rounds regardless of the time elapsed after initial intervention (χ^2^ = 3.74, df = 3, P>0.25), treatment coverage (i.e., community-wide versus selective, χ^2^ = 3.20, df = 2, P>0.20), and insecticide dose (standard versus double dose, χ^2^ = 1.73, df = 1, P>0.15). Persistent infestations were detected 4–5 months after selective applications of pyrethroids with standard dose in 2 (4%) of 55 infested sites, and with a double dose in 3 (10%) of 30 infested sites. In sites negative before selective applications, infestations were subsequently detected in 1 of 21 sites sprayed with a standard dose, and in none of 9 sites sprayed with a double dose.

Regarding the effectiveness of selective treatments at house-compound level, TMC searches found persistent infestations (in at least one site per house) in 13–37% of the treated houses within 4–6 months after each of the four selective spray rounds (Figure 2B). No significant differences in effectiveness between spray rounds were detected despite variations in treatment criteria (χ^2^ = 3.70, df = 3, P>0.30). Infestation persisted to the subsequent survey conducted 4–5 months later in 10% of the 31 infested houses that were fully sprayed with a double dose of pyrethroids at 17 MPS, whereas none of the 292 negative houses not sprayed with pyrethroids at that time had a subsequent infestation.

**Figure 3:**
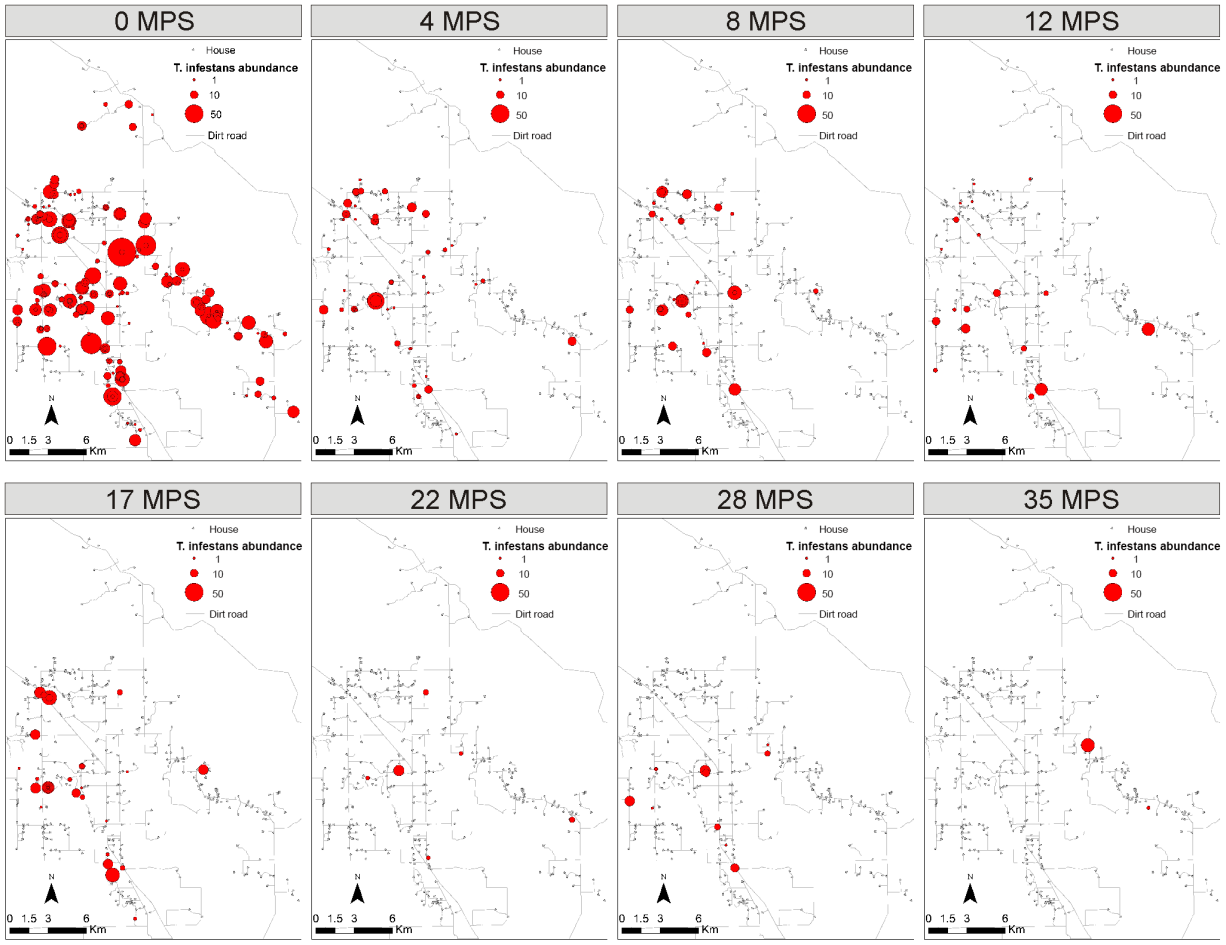
Spatio-temporal distribution of the abundance of *T. infestans* per site as determined by timed-manual collections. Pampa del Indio, 2007–2010. MPS: months postspraying. Dotted line at 4 MPS divides the western and eastern sections of the study area.

**Figure 4:**
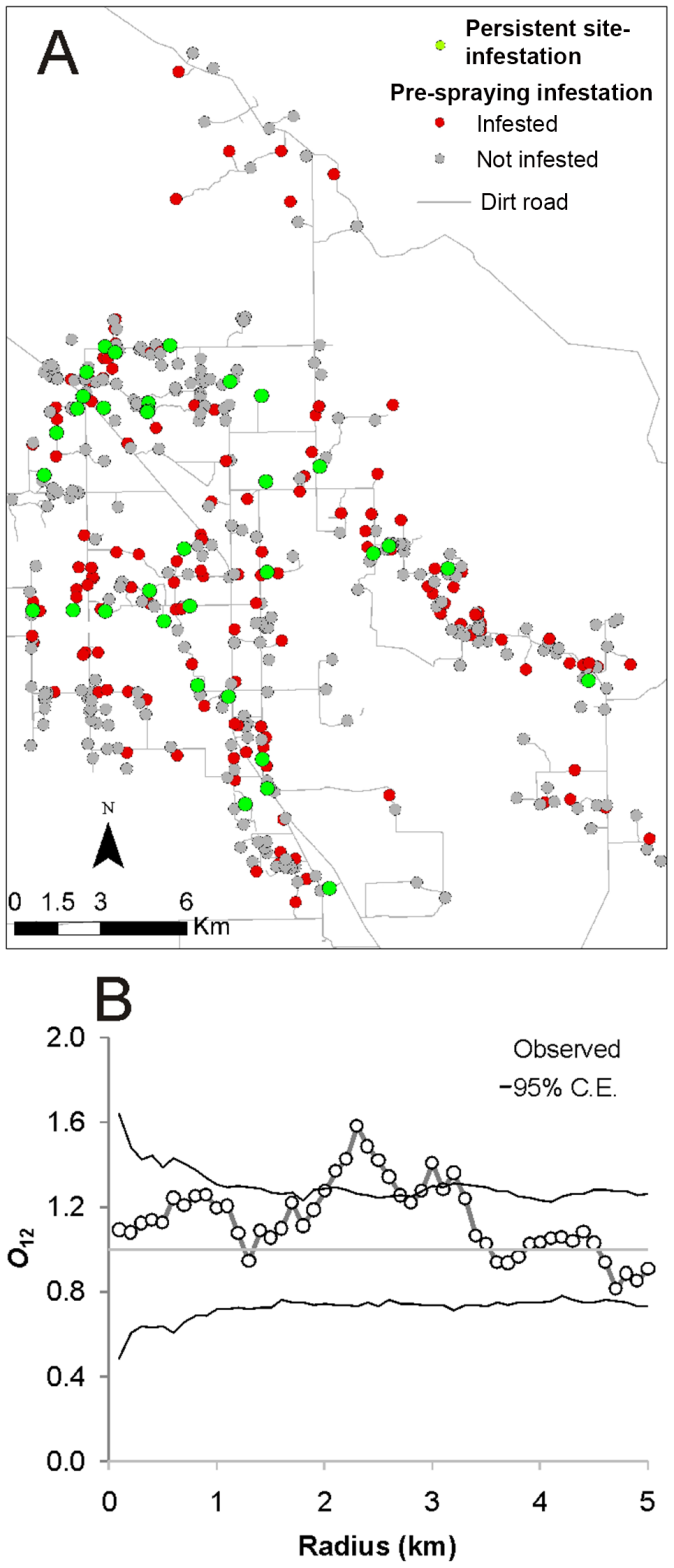
Distribution of houses infested with *T. infestans* before and 4 months after initial interventions. Pampa del Indio, 2007–2008. A: Map showing the location of persistently infested houses. B: Spatial analysis of persistently infested houses with respect to prespraying infested houses. C.E.: confidence envelope according to null model.

The impacts of the initial community-wide pyrethroid spray on house infestation were not homogeneous across the study area (Figure 3). Houses with a persistent infestation at 4 MPS were spatially aggregated up to a distance of 2.5 km from houses infested at 0 MPS (Figure 4). Although the western and eastern sections had similar house infestation prevalence at 0 MPS (50.9% and 46.9%, respectively; Fisher's exact test, P = 0.49), infestation at 4 MPS in the western section (15.8%) was three times higher than in the eastern section (4.9%; Fisher's exact test, P = 0.005) (Figure 3). Considering the entire follow-up period, most (76%) of the infestations detected after initial intervention occurred in houses that had been infested before community-wide spraying with insecticides. When houses with a putative persistent infestation were excluded from consideration, postspraying infestation was still significantly more frequent among houses infested before initial spraying (17 of 122, 13.9%) than among those that had not been infested at baseline (10 of 190, 5.3%) (Fisher's exact test, P<0.001).

Of the eight sites (each in a different house) infested with *T. infestans* at 22 MPS that were immediately sprayed with malathion, two were infested at 28 MPS (see Text S1 for further details on apparent rainstorm effects). At 28 MPS, the 10 sites found infested (at 10 houses) were sprayed with malathion and none of them were found infested at 35 MPS.

### Infestation and related factors

**Figure 5:**
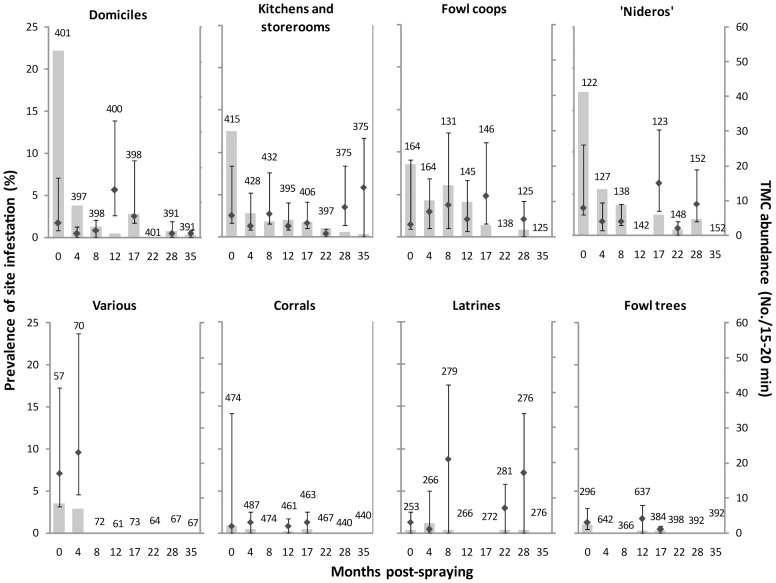
Prevalence and abundance of site-specific infestation with *T. infestans* according to main type of ecotope. Pampa del Indio, 2007–2010. Infestations assessed by timed-manual collections (TMC). Only site-level data from the main ecotopes (domiciles, kitchens or storerooms, fowl coops and ‘nideros’) are included. Domiciliary sites numbered 401 because a given house compound may have more than one domiciliary site, in an analog fashion to peridomestic sites. Numbers above bars indicate number of sites inspected. Symbols indicate median bug abundance by TMC in infested sites; whiskers represent the range between the first and third quartiles.

**Figure 6:**
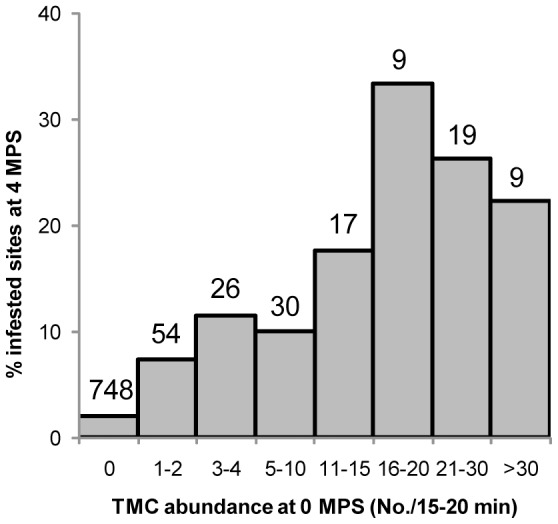
Prevalence of site-level infestation with *T. infestans* at 4 MPS according to prespraying bug abundance. Pampa del Indio, 2007–2008. Infestation assessed by timed-manual collections (TMC). Only data from the main ecotopes (domiciles, kitchens or storerooms, fowl coops and ‘nideros’) are included. Numbers above bars indicate the number of sites within each category.

Peridomestic infestation prevalence exceeded that in domestic sites during the entire follow-up after initial interventions, even though prespraying infestation was slightly higher in domiciles (Figure 1). The most frequently infested ecotopes before the initial community-wide spraying with pyrethroids were also the ones most frequently infested after selective treatments, including domiciles, kitchens or storerooms, fowl coops and ‘nideros’ –an elevated shelf made of wood or sometimes bricks where chickens, and occasionally turkeys or ducks, nested (Figure 5). Corrals and other types of ecotope (latrines, ovens, trees with chickens, and others) were rarely infested. The relative frequency of infested sites at 4 MPS increased with increasing bug abundance determined by TMC before initial interventions (Figure 6).

The reported use of insecticides by householders varied significantly from 41.4% to 69.0% during the follow-up (Table S2) (χ^2^ = 63.14, df = 4, P<0.0001). These variations were mainly caused by an increase in the number of households using domestic insecticide aerosols at least every two months. Householders reported applying insecticides in domiciles, kitchens and storerooms.

The results of the multi-model inference analysis of factors putatively related to new infestations at the first year of interventions or subsequently are presented in Table 2. Refuge availability showed the highest RI (0.98) followed by distance to the nearest site found infested at the preceding survey (RI = 0.81) and, much less important, by distance to the nearest site found infested two surveys earlier (RI = 0.60). Reported insecticide use had a low RI (0.39). The chance of new infestations increased with more refuges for bugs and with more proximity to the nearest infested site at the preceding surveys.

**Table 2:** Relative importance (RI) and effects of variables in relation to site-level infestation with *T. infestans* at 12–35 MPS using a multi-model inference approach.

	Infestations		
Variable	RI	OR	S.E.
Refuge availability	0.98	1.72	0.74
Distance at *t*−1	0.81	0.79	0.28
Distance at *t*−2	0.60	0.85	0.27
Reported insecticide use	0.39		
No		1.00	
Yes		1.16	0.40

Only sites from houses uninfested at 4 or 8 MPS are considered. ‘Distance’ refers to the distance (in m) to the closest infested site at the preceding survey (‘*t*−1’) or at ‘*t*−2’. The RI of variables was assessed by multi-model inference comparisons based on Akaike Information Criterion. OR: odds ratio; S.E.: standard error.

TMC significantly outperformed householders' bug detection before and after interventions except in domiciles after initial insecticide spraying (Table 3). However, householders contributed to enhanced detection of *T. infestans* by capturing and handing bugs on to the research team in 108 occasions, 66% of which occurred in sites negative by TMC at the survey that immediately followed householders' collections. Conversely, TMC detected *T. infestans* bugs in 328 occasions, 89% of which occurred in the absence of householders' collections. Householders collected only one adult *T. infestans* in 48 (44%) of the occasions; >1 adult in 50 (46%) occasions, and nymphs in 40 (37%) of their bug collections. Householders captured bugs most frequently in domiciles (72 of 108), followed by kitchens, storerooms and ‘nideros’.

**Table 3:** Comparison between householders' and timed-manual collections of *T. infestans* according to ecotope and survey.

Ecotope	Months post spraying	TMC/householders' collections				Total	Mc Nemar test
		+/+	+/−	−/+	−/−		
Domiciles	0	13	76	13	299	401	P<0.001
	4–35	8	29	38	2701	2776	P>0.1
Kitchens and storerooms	0	5	47	0	363	415	P<0.001
	4–35	5	37	11	2755	2808	P<0.001
Other*	0	0	46	4	1565	1615	P<0.001
	4–35	6	56	5	13008	13075	P<0.001
Total		37	291	71	20704	21103	

### Simulation of infestation under alternative spray criteria

**Figure 7:**
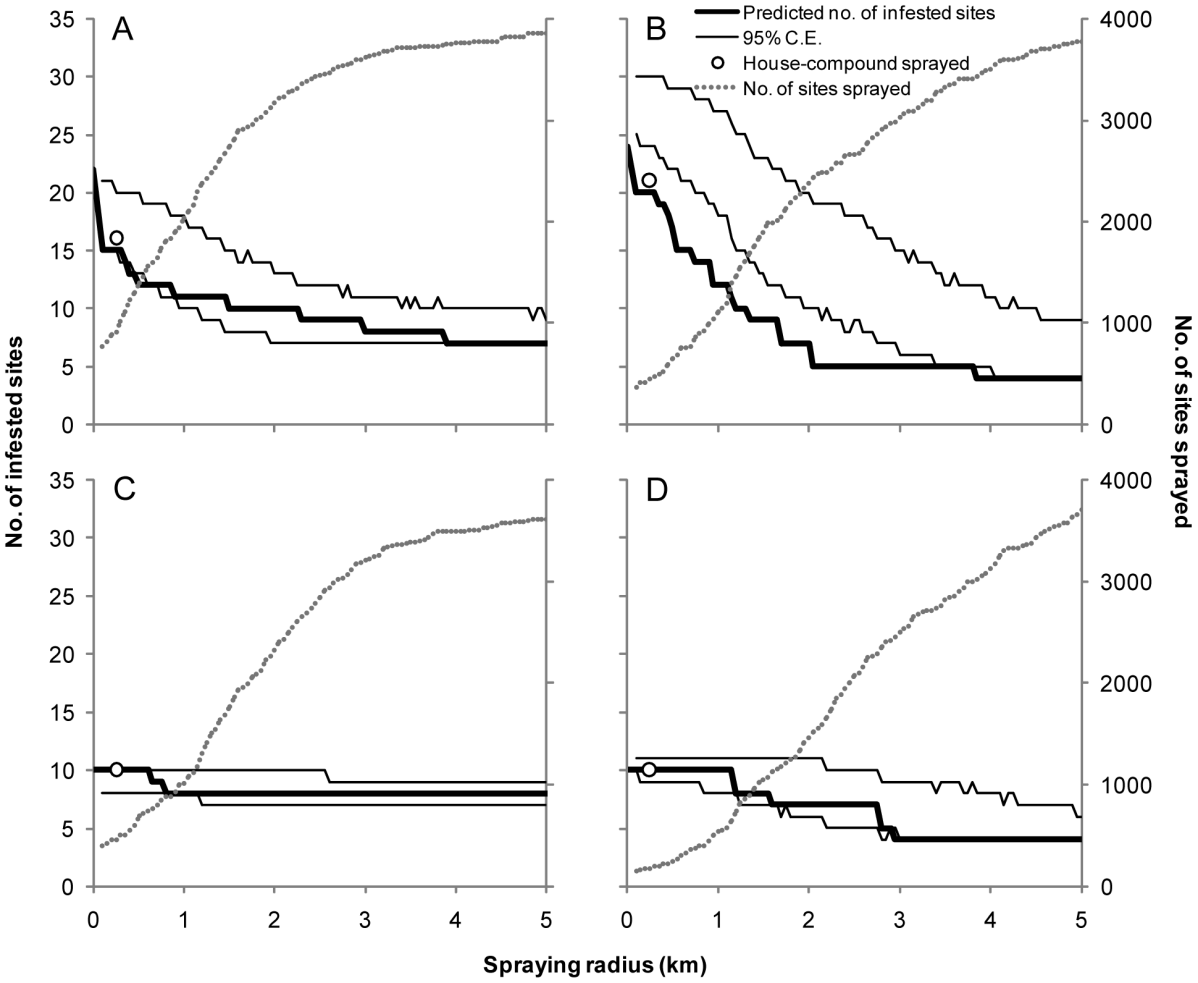
Predicted infestation under different criteria of selective sprays with insecticides. Each panel presents the expected outcome at the survey subsequent to the selective spray. A: 12 MPS. B: 17 MPS. C: 22MPS. D: 28 MPS. Bold line represents the predicted infestation as a function of the radius of insecticide spray coverage around each of the detected foci. Thin lines represent the 95% confidence envelope (C.E.) of predicted values. Circles indicate the predicted infestation if the entire house compound had been sprayed. Dotted lines represent the number of hypothetically sprayed sites as a function of spray coverage radius.

For each of the first four selective spray rounds (8–22 MPS) we assessed the hypothetical effects of spraying all sites within a house compound that at least had an infested site, and spraying all sites within a given radius around the detected focus. The number of hypothetically infested sites declined gradually up to a radius of 2–3 km while the total number of hypothetically sprayed sites increased considerably (Figure 7). Such decline in infested sites exceeded that expected by chance up to 0.5 km (∼1,500 sites sprayed) for the first selective spray round (Figure 7A) and up to 2–3 km (∼2,300 sites sprayed within 2 km) for the second round (Figure 7B). Decreases in the number of hypothetically infested sites in the two subsequent rounds –when the entire infested house compounds were actually sprayed– were within the confidence envelope expected by chance (Figure 7C,D).

### Qualitative analysis of study cases

Overall figures of the impacts of insecticide spraying on infestation provide a broad picture while hiding some relevant observations. Below we report on several specific cases (sites or houses), and relate their infestation status to defined events or processes:

- Recurrent infestations at house-compound level detected two surveys after selectively spraying infested sites only: Of 19 house compounds with ≥1 site selectively sprayed and with no site found infested in the subsequent survey, 11 house compounds were infested again two surveys later (i.e., at t + 2, 9–10 months after the first selective spray)(Figure S1).
- Persistent infestations at house-compound level after selectively spraying infested sites were suppressed only after spraying the entire house compound: After selective treatment of infested sites only, 10 house compounds had one or more sites infested in the subsequent survey (Figure S1). Six of them ceased to be infested after spraying of the complete house compound with pyrethroids at 17 MPS.
- Persistent infestations after spraying the entire house compound: Three house compounds infested and re-sprayed with pyrethroids at 17 MPS were still infested at 22 MPS: two had a persistent infestation at site level, one of them caused by pyrethroid resistance (one of the experimental sites reported elsewhere [35]) and the other probably caused by moderate insecticide resistance combined with exposure to weather events. The third house compound had a newly infested site, different from the one found infested at 17 MPS. Further details on the limitations of pyrethroid sprays in suppressing local infestations and on householders' behavior in response to infestation are provided in Text S1.

## Discussion

Our results show that although house infestation was dramatically reduced after initial interventions, the local elimination of *T. infestans* was not achieved even after three years of intensified vector surveillance and frequent selective insecticide sprays conducted by professional personnel under close supervision. The local occurrence of bug populations with moderate resistance to pyrethroid insecticides [35] and the limited effectiveness of selective treatment of infested sites contributed to the challenging scenario emerging in the Gran Chaco.

The full-coverage pyrethroid spraying did not reach the expected target of vector control programs (<5% of infested houses within 6–12 MPS, to account for technical errors including suboptimal spray coverage) despite the occurrence of lower baseline infestation levels than in other similar rural areas with no recent vector control actions [11], [27], [28], [41], [42]. Subsequent selective sprays with pyrethroids did not perform better, and its effectiveness remained approximately stable across several spray rounds (Figure 2) despite bug colonies had been decimated. Unlike in previous trials [21], [22], both simple- and double-dose applications had similarly limited effects consistent with the occurrence of diminished susceptibility to pyrethroid insecticides [35].

We recorded a large degree of spatial and temporal heterogeneity in infestation, the apparent effectiveness of insecticide spraying, and people's practices related to infestation (Text S1). The spatial distribution of pyrethroid resistance was apparently aggregated, as determined from the occurrence of early persistent infestations. None of these facts would have been noticed without frequent monitoring of infestations at site level and upscale. Few vector control failures associated with pyrethroid resistance have been reported [30], [31], [35], whereas the number of resistant *T. infestans* populations scattered through most of its current distribution range has gradually increased [32]. Alerted by our findings, in 2010 the Chagas vector control program detected a new focus of high pyrethroid resistance associated with repeated control failures at 100 km from our study area [43], in a district which also had a history of sporadic house spraying with insecticides. Adequate monitoring of treatment effects in routine operations of vector control programs may reveal hitherto unknown resistant foci and the actual effectiveness of control interventions.

Selective pyrethroid sprays of sites infested only performed poorly. Frequently the treated bug colonies were apparently suppressed, yet in a few months other infestations became detectable in adjacent sites within the same house compound (Figure S1), either because they had not previously been detected by TMC or because bugs dispersed actively from the detected foci around the time of selective treatment. In our study, the multi-model based association between new site-level infestations and the proximity of other foci detected at one or two preceding surveys is consistent with: (i) spatial aggregation of *T. infestans* foci occurring at various scales [21], [44]–[46]; ii) a six-month time lag between detection of foci and dispersal events of *T. infestans* inferred from the spatio-temporal dynamics of reinfestation patterns [47], [48], and iii) frequent flight or walking dispersal of *T. infestans* during spring-summer [e.g., 49]. These findings support the extension of spray coverage to all sites within the house compound and the consideration of the compound as the minimal vector control unit, rather than the individual infested sites.

The simulation results further suggest that enhanced bug control would be achieved if all suitable sites within 500–1,000 m of the detected foci were sprayed. Similarly, a longitudinal study conducted in the Argentinean dry Chaco reported significant spatial aggregation of reinfested sites at 25–500 m around residual foci, and recommended extending selective insecticide sprays up to a distance of 450–500 m around the detected foci [21], [44]. This tactic (justifiable if the goal were vector elimination) implies a substantial increase in the frequency of sites sprayed and the resources needed. Therefore, its relative merits must be framed within the stringent operational and economic constraints of vector control programs in the study region; variations in the spatial layout of villages (i.e., connectivity), and eventual landscape effects on vector dispersal (e.g., barriers).

Refuge availability, the main bug habitats, and prespraying bug abundance were closely related to postspraying site-level infestation after initial interventions, in agreement with existing evidence [35]. The multi-model inference approach showed that refuge availability was highly important for explaining variations in site infestation before and after interventions. Similarly, nearly all detected infestations occurred in the same four key ecotopes before and after interventions. Moreover, peridomestic ecotopes were more frequently infested than domiciles after initial interventions –a recurrent pattern related to the higher exposure of peridomestic structures (especially chicken coops and ‘nideros’) to sunlight, rain and dust, all of which undermine the activity of pyrethroids [22], [24], [25]. The relative occurrence of a persistent site-level infestation at 4 MPS increased substantially with increasing prespraying bug abundance, as recorded elsewhere [22], [24], [25], [50]. Conversely, reported insecticide use by householders had a low RI for explaining new infestations despite it was closely associated with prespraying infestation in domiciles, kitchens and storerooms [33]. Such differences are probably related to the few domiciles, kitchens and storerooms found infested at 12 MPS or subsequently. Taken together, these results are highly relevant for improved vector control and imply that: i) factors with substantial effects on infestation remain approximately stable before and after insecticide spraying (e.g., refuge and host availability), and ii) prespraying data on the main types of infested ecotopes and bug abundance provide valuable information on the future effectiveness of insecticide spraying and may be used for identifying sites, houses or village sections most likely to be problematic for vector control.

House infestation prevalence fell below 1% only after multiple inspection and selective spray rounds with pyrethroids and finally, selective treatment of highly persistent foci with malathion. During the follow-up, several other local events potentially affecting infestation may have confounded the specific effects of insecticide spraying. Some of the cases analyzed (Text S1) illustrate modifications introduced by householders (e.g., physical structure of sites, removal of infested sites, host management, and non-professional insecticide use) combined with adverse effects related to occasional rainstorms, operational problems during insecticide application (e.g., sites difficult to spray adequately, as in a storeroom full of corn, or errors in procedures or planning), and imperfect detection methods [41], [51]–[53]. These factors may enhance or diminish substantially the effects of insecticide spraying.

Timed manual searches conducted by skilled personnel using a dislodging agent is the standard method used to assess infestations in intervention trials despite its limited sensitivity and precision, especially at low bug densities [8], [35], [51], [53]. In our study, its shortcomings were partially compensated by recurrent, very frequent searches of bugs in identified sites (averaging approximately one person-hour per house compound) and promotion of householders' bug collections. The overall frequency of infestations detected by TMC (and bug catches) was much larger than those achieved by householders, unlike in other settings with lower bug densities [51], [54]. Local villagers were aware that insecticidal treatments would continue regardless of their compliance with capturing and keeping the bugs, and therefore may have been less motivated to do so. Community-based vector surveillance has played an increasing role over recent decades [8], [24], [42], [55], [56], yet the ability of householders to detect bugs is widely variable depending on various factors [8], [51], [57] and may be more difficult to standardize. Householders detected proportionally more infestations in domestic rather than peridomestic ecotopes [51], perhaps because they were more motivated to suppress bugs from sleeping quarters or were there when bugs emerged from refuges. They also detected several infestations missed by subsequent TMC searches, several of which may have been recent invasions (not established bug colonies). More attention needs to be given to vector surveillance in peridomestic sites either through appropriate training of rural villagers or using baited traps [14].

One limitation of our study is that we have not assessed the impact of interventions on parasite transmission, as vector-borne transmission of *T. cruzi* to humans and dogs may occur at very low infected-bug densities in high-risk areas [58]. The use of a house infestation prevalence of 5% as a threshold for parasite transmission mediated by *T. infestans* “is not supported by rigorous evidences but rather derived from data on *Triatoma infestans* in Brazil without scientific justification” [59]. The validity of the refuge availability index and other predictors was discussed before [35]. Although most of the bugs collected after interventions most likely survived treatment at site level (i.e., residual foci), immigrant bugs from other sources may explain in part new infestations. Use of microsatellite markers and wing geometric morphometry [e.g.], [ 12,18] may provide concluding evidence on their relative contribution. A major strength of our intervention trial was the detailed information collected systematically at site level in a sizable number of house compounds every 4–7 months over a three-year period.

### Implications for policy

Evidence of the obstacles to suppress *T. infestans* in the Gran Chaco ecoregion [e.g.], [ 11], [21,28] led the Southern Cone Initiative to turn from the initial goal of vector elimination into the less ambitious one of controlling house infestations and interrupting vector- and blood-borne transmission in recent years [60]. Our current results document substantial geographic variations in the characteristics of persistent foci in the region [21], [28], [29], [33], and provide guidance on the effort levels needed to suppress *T. infestans* in an area with moderate pyrethroid resistance. The chronic limitations in personnel and resources in the Latin American health sector (more so in rather remote rural areas) pose serious obstacles to vector and disease suppression efforts. Research on the real functioning of disease control programs and their capacity to operate and modify what they do and how they do it is needed to push knowledge closer to its effective application [61], [62]. The ultimate limitations of insecticide-based control strategies are that they do not change the material conditions that favor the occurrence and spread of domestic vectors [63] such as *T. infestans*, and the eventual emergence of insecticide resistance. In addition to careful, systematic residual insecticide applications, our findings confirm that housing modifications and development policies that improve material conditions of rural villagers and reduce habitat suitability for *T. infestans* [35] may contribute substantially to sustainable vector and disease control in the Gran Chaco.

## Supporting Information

Figure S1 **Examples to illustrate persistent infestations with** ***T. infestans*** **at house-compound level.** Each row corresponds to a georeferenced (identifiable) site. Only sites ever found infested at least once in the selected houses are shown. Colors indicate infestation status at each house and survey according to collection method.
(DOC)

Table S1 **Number of houses inhabited, inspected by timed manual collections (TMC) and sprayed with insecticides in Pampa del Indio.**
(DOC)

Table S2 **Insecticide use between successive surveys as reported by householders.**
(DOC)

Text S1 **Details on relevant cases regarding insecticide spraying effectiveness and householders' practices.**
(DOC)

## References

[1] Dias E, Pellegrino J (1948) Alguns ensaios com o “Gammexane” no combate aos transmissores da doença de Chagas. Brasil Med 62: 185–191.

[2] Romaña C, Abalos JW (1948) Acción del “Gammexane” sobre los triatomideos. Control domiciliario. An Inst Med Reg Tucumán 2: 95–106.

[3] Dias JCP, Silveira AC, Schofield CJ (1999) The evolution of Chagas disease (American trypanosomiasis) control after 90 years since Carlos Chagas discovery. Mem Inst Oswaldo Cruz 94: 103–121. 10.1590/S0074-02761999000700011 10677697

[4] Silveira AC [ed.] (2002) El control de la enfermedad de Chagas en los países del Cono Sur de América. Historia de una iniciativa internacional. 1991/2001. Uberaba: Facultad de Medicina, Pan American Health Organization.

[5] World Health Organization (2007) Reporte sobre la enfermedad de Chagas. TDR/SWG/09. Geneva: WHO.

[6] Feliciangeli MD, Campbell-Lendrum DH, Martínez C, González D, Coleman P, et al (2003) Chagas disease control in Venezuela: lessons for the Andean region and beyond. Trends Parasitol 19: 44–49. 12488226 10.1016/s1471-4922(02)00013-2

[7] Schofield CJ, Jannin J, Salvatella R (2006) The future of Chagas disease control. Trends Parasitol 22: 583–588. 17049308 10.1016/j.pt.2006.09.011

[8] Abad-Franch F, Vega MC, Rolón MS, Santos WS, Rojas de Arias A (2011) Community participation in Chagas disease vector surveillance: systematic review. PLoS Negl Trop Dis 5: e1207. 21713022 10.1371/journal.pntd.0001207 PMC3119642

[9] Soler CA, Schenone H, Reyes H (1969) Problemas derivados de la reaparición de *Triatoma infestans* en viviendas desinsectadas y el concepto de reinfestación. Bol Chil Parasitol 24: 83–87. 4904048

[10] Noireau F, Rojas Cortez M, Monteiro F, Jansen A, Torrico F (2005) Can wild *Triatoma infestans* foci in Bolivia jeopardize Chagas disease control efforts? Trends Parasitol 21: 1–12. 15639733 10.1016/j.pt.2004.10.007

[11] Gürtler RE, Kitron U, Cecere MC, Segura EL, Cohen JE (2007) Sustainable vector control and management of Chagas disease in the Gran Chaco, Argentina. Proc Natl Acad Sci USA 104: 16194–16199. 17913895 10.1073/pnas.0700863104 PMC2042184

[12] Ceballos LA, Piccinali RV, Marcet PL, Vazquez-Prokopec GM, Cardinal MV, et al (2011) Hidden sylvatic foci of the main vector of Chagas disease *Triatoma infestans*: threats to the vector elimination campaign? PLoS Negl Trop Dis 5: e1349. 22039559 10.1371/journal.pntd.0001365 PMC3201917

[13] Buitrago R, Waleckx E, Bosseno MF, Zoveda F, Vidaurre P, et al (2010) First report of widespread wild populations of *Triatoma infestans* (Reduviidae, Triatominae) in the valleys of La Paz, Bolivia. Am J Trop Med Hyg 82: 574–579. 20348501 10.4269/ajtmh.2010.09-0325 PMC2844558

[14] Rojas de Arias A, Abad-Franch F, Acosta N, López E, González N, et al (2012) Post-control surveillance of *Triatoma infestans* and *Triatoma sordida* with chemically-baited sticky traps. PLoS Negl Trop Dis 6: e1822. 23029583 10.1371/journal.pntd.0001822 PMC3441417

[15] Diotaiuti L, Faria Filho O, Carneiro FCF, Dias JCP, Pires HHR, et al (2000) Aspectos operacionais do controle do *Triatoma brasiliensis* . Cad Saude Publ 16: 61–67. 11119320

[16] Abad-Franch F, Paucar CA, Carpio CC, Cuba Cuba CA, Aguilar VHM, et al (2001) Biogeography of Triatominae (Hemiptera: Reduviidae) in Ecuador: implications for the design of control strategies. Mem Inst Oswaldo Cruz 96: 611–620. 11500757 10.1590/s0074-02762001000500004

[17] Cuba Cuba CA, Abad-Franch F, Roldán Rodríguez J, Vargas Vásquez F, Pollack Velásquez L, et al (2002) The triatomines of northern Peru, with emphasis on the ecology and infection by trypanosomes of *Rhodnius ecuadoriensis* (Triatominae). Mem Inst Oswaldo Cruz 97: 175–183. 12016438 10.1590/s0074-02762002000200005

[18] Dumonteil E, Tripet F, Ramírez-Sierra MJ, Payet V, Lanzaro G, et al (2007) Assessment of *Triatoma dimidiata* dispersal in the Yucatan Peninsula of Mexico by morphometry and microsatellite markers. Am J Trop Med Hyg 76: 930–937. 17488918

[19] The Nature Conservancy, Fundación Vida Silvestre Argentina, Fundación para el Desarrollo Sustentable del Chaco, and Wildlife Conservation Society Bolivia(2005) Evaluación Ecorregional del Gran Chaco Americano/Gran Chaco Americano Ecoregional Assessment. Buenos Aires: Fundación Vida Silvestre Argentina.

[20] Gürtler RE (2009) Sustainability of vector control strategies in the Gran Chaco region: current challenges and possible approaches. Mem Inst Oswaldo Cruz 104 Suppl. 1 52–59. 19753458 10.1590/s0074-02762009000900009 PMC3072747

[21] Cecere MC, Vazquez-Prokopec GM, Gürtler RE, Kitron U (2004) Spatio-temporal analysis of reinfestation by *Triatoma infestans* (Hemiptera: Reduviidae) following insecticide spraying in a rural community in northwestern Argentina. Am J Trop Med Hyg 71: 803–810. 15642975 PMC1351234

[22] Gürtler RE, Canale DM, Spillmann C, Stariolo R, Salomón OD, et al (2004) Effectiveness of residual spraying of peridomestic ecotopes with deltamethrin and permethrin on *Triatoma infestans* in rural western Argentina: a district-wide randomized trial. Bull World Health Organ 82: 196–205. 15112008 PMC2585938

[23] Porcasi X, Catalá SS, Hrellac H, Scavuzzo MC, Gorla DE (2006) Infestation of rural houses by *Triatoma infestans* (Hemiptera: Reduviidae) in southern area of Gran Chaco in Argentina. J Med Entomol 43: 1060–1067. 17017246 10.1603/0022-2585(2006)43[1060:iorhbt]2.0.co;2

[24] Cecere MC, Vazquez-Prokopec GM, Ceballos LA, Gurevitz JM, Zárate JE, et al (2006) Comparative trial of effectiveness of pyrethroid insecticides against peridomestic populations of *Triatoma infestans* in northwestern Argentina. J Med Entomol 43: 902–909. 17017227 10.1603/0022-2585(2006)43[902:ctoeop]2.0.co;2 PMC1894891

[25] Cecere MC, Vazquez-Prokopec GM, Ceballos LA, Boragno S, Zárate JE, et al (2013) Improved chemical control of Chagas disease vectors in the dry Chaco region. J Med Entomol 50: 394–403. 23540129 10.1603/me12109 PMC3773707

[26] Guillén E (2002) El control de la enfermedad de Chagas en Bolivia. In: Silveira AC, editor. El control de la enfermedad de Chagas en los países del Cono Sur de América. Historia de una iniciativa internacional. 1991/2001. Uberaba: Facultad de Medicina, Pan American Health Organization.

[27] Segura EL (2002) El control de la enfermedad de Chagas en la República Argentina. In: Silveira AC, editor. El control de la enfermedad de Chagas en los países del Cono Sur de América. Historia de una iniciativa internacional. 1991/2001. Uberaba: Facultad de Medicina, Pan American Health Organization.

[28] Canale DM, Carcavallo RU (1985) *Triatoma infestans* (Klug). In: Carcavallo RU, Rabinovich JE, Tonn RJ, editors. Factores biológicos y ecológicos en la Enfermedad de Chagas. Buenos Aires: Ministerio de Salud y Acción Social. pp. 237–250.

[29] Gorla DE, Schofield CJ (1989) Population dynamics of *Triatoma infestans* under natural climatic conditions in the Argentine Chaco. Med Vet Entomol 3: 179–194. 2519662 10.1111/j.1365-2915.1989.tb00497.x

[30] Picollo MI, Vassena C, Orihuela PS, Barrios S, Zaidemberg M, et al (2005) High resistance to pyrethroid insecticides associated with ineffective field treatments in *Triatoma infestans* (Hemiptera: Reduviidae) from northern Argentina. J Med Entomol 42: 637–642. 16119553 10.1093/jmedent/42.4.637

[31] Vassena C, Picollo MI, Orihuela PS, Zerba E (2006) Desarrollo y manejo de la resistencia a insecticidas. In: Rojas Cortez M, editor. Triatominos de Bolivia y la enfermedad de Chagas. La Paz: Ministerio de Salud y Deportes, Bolivia.

[32] Lardeux F, Depickere S, Duchon S, Chávez T (2010) Insecticide resistance of *Triatoma infestans* (Hemiptera, Reduviidae) vector of Chagas disease in Bolivia. Trop Med Int Health 15: 1037–1048. 20545921 10.1111/j.1365-3156.2010.02573.x

[33] Gurevitz JM, Ceballos LA, Gaspe MS, Alvarado-Otegui JA, Enríquez GF, et al (2011) Factors affecting infestation by *Triatoma infestans* in a rural area of the humid Chaco in Argentina. PLoS Negl Trop Dis 5: e1349. 22028941 10.1371/journal.pntd.0001349 PMC3196485

[34] Alvarado-Otegui JA, Ceballos LA, Orozco MM, Enríquez G, Cardinal MV, et al (2012) The sylvatic transmission cycle of *Trypanosoma cruzi* in the humid Chaco of Argentina. Acta Trop 124: 79–86. 22771688 10.1016/j.actatropica.2012.06.010 PMC3444808

[35] Gurevitz JM, Gaspe MS, Enríquez GF, Vassena CV, Alvarado-Otegui JA, et al (2012) Unexpected failure to control Chagas disease vectors with pyrethroid spraying in northern Argentina. J Med Entomol 49: 1379–1386. 23270166 10.1603/me11157 PMC3760256

[36] Burnham KP, Anderson DR (2002) Model selection and multimodel inference: a practical information-theoretic approach. New York: Springer-Verlag.

[37] R Development Core Team (2008) R: A language and environment for statistical computing computer program, version 2.13.0. Vienna.

[38] Stoyan D, Stoyan H (1994) Fractals, random shapes and point fields. Methods of geometrical statistics. New York: John Wiley & Sons.

[39] Wiegand T, Moloney KA (2004) Rings, circles, and null-models for point pattern analysis in ecology. Oikos 104: 209–229.

[40] The MathWorks I (2006) Matlab Version 7.3.0 (R2006b) computer program. Natick: The MathWorks.

[41] Chuit R, Paulone I, Wisnivesky-Colli C, Bo R, Pérez AC, et al (1992) Results of a first step toward community-based surveillance of transmission of Chagas' disease with appropriate technology in rural areas. Am J Trop Med Hyg 46: 444–450. 1575292 10.4269/ajtmh.1992.46.444

[42] Guillén G, Jemio A, Alfred Cassab J, Teixeira Pinto C, Schofield CJ (1997) Chagas disease vector control in Tupiza, Southern Bolivia. Mem Inst Oswaldo Cruz 92: 1–8. 10.1590/s0074-02761997000100001 9302405

[43] Carvajal G, Mougabure-Cueto G, Toloza AC (2012) Toxicity of non-pyrethroid insecticides against *Triatoma infestans* (Hemiptera: Reduviidae). Mem Inst Oswaldo Cruz 107: 675–679. 22850959 10.1590/s0074-02762012000500015

[44] Cecere MC, Vazquez-Prokopec GM, Gürtler RE, Kitron U (2006) Reinfestation sources for Chagas disease vector, *Triatoma infestans*, Argentina. Emerg Infect Dis 12: 1096–1102. 16836826 10.3201/eid1207.051445 PMC1853288

[45] Levy MZ, Quíspe-Machaca VR, Ylla-Velásquez JL, Waller LA, Richards JM, et al (2008) Impregnated netting slows infestation by *Triatoma infestans* . Am J Trop Med Hyg 79: 528–534. 18840739 PMC2659296

[46] Vazquez-Prokopec GM, Spillmann C, Zaidenberg M, Gürtler RE, Kitron U (2012) Spatial heterogeneity and risk maps of community infestation by *Triatoma infestans* in rural northwestern Argentina. PLoS Negl Trop Dis 6: e1178. 10.1371/journal.pntd.0001788 PMC3419179 22905276

[47] zu Dohna H, Cecere MC, Gürtler RE, Kitron U, Cohen JE (2007) Re-establishment of local populations of vectors of Chagas disease after insecticide spraying. J Appl Ecol 44: 220–227. 17710182 10.1111/j.1365-2664.2006.01243.x PMC1948873

[48] zu Dohna H, Cecere MC, Gürtler RE, Kitron U, Cohen JE (2009) Spatial re-establishment dynamics of local populations of vectors of Chagas disease. PLoS Negl Trop Dis 3: e490. 19636363 10.1371/journal.pntd.0000490 PMC2709728

[49] Vazquez-Prokopec GM, Ceballos LA, Marcet PL, Cecere MC, Cardinal MV, et al (2006) Seasonal variations in active dispersal of natural populations of *Triatoma infestans* in rural north-western Argentina. Med Vet Entomol 20: 1–6. 17044877 10.1111/j.1365-2915.2006.00637.x PMC1894892

[50] Gürtler RE, Petersen RM, Cecere MC, Schweigmann NJ, Chuit R, et al (1994) Chagas disease in north-west Argentina: risk of domestic reinfestation by *Triatoma infestans* after a single community-wide application of deltamethrin. Trans R Soc Trop Med Hyg 88: 27–30. 8153989 10.1016/0035-9203(94)90483-9

[51] Gürtler RE, Cecere MC, Canale DM, Castañera MB, Chuit R, et al (1999) Monitoring house reinfestation by vectors of Chagas disease: a comparative trial of detection methods during a four-year follow-up. Acta Trop 72: 213–234. 10206120 10.1016/s0001-706x(98)00096-5

[52] Abad-Franch F, Ferraz G, Campos C, Palomeque FS, Grijalva MJ, et al (2010) Modeling disease vector occurrence when detection is imperfect: infestation of Amazonian palm trees by triatomine bugs at three spatial scales. PLoS Negl Trop Dis 4: e620. 20209149 10.1371/journal.pntd.0000620 PMC2830460

[53] Gürtler RE, Chuit R, Cecere MC, Castañera MB (1995) Detecting domestic vectors of Chagas disease: a comparative trial of six methods in north-west Argentina. Bull World Health Organ 73: 487–494. 7554021 PMC2486773

[54] Silva RA, Bonifácio PR, Wanderley DMV (1999) Doença de Chagas no Estado de São Paulo: comparação entre pesquisa ativa de triatomíneos em domicílios e notificação de sua presença pela população em área sob vigilancia entomológica. Rev Soc Bras Med Trop 32: 653–659. 10881102 10.1590/s0037-86821999000600007

[55] Garcia-Zapata MT, Marsden PD (1993) Chagas' Disease: control and surveillance through use of insecticides and community participation in Mambaí, Goiás, Brazil. Bull PAHO 27: 265–279. 8220521

[56] Dias JCP (2002) O controle da doença de Chagas no Brasil. In: Silveira AC, editor. El control de la enfermedad de Chagas en los países del Cono Sur de América. Historia de una iniciativa internacional. 1991/2001. Uberaba; Faculta de Medicina, Pan American Health Organization.

[57] Dias JCP (1991) Chagas disease control in Brazil: which strategy after the attack phase? Ann Soc Belge Med Trop 71: 75–86. 1793283

[58] Gürtler RE, Cecere MC, Lauricella MA, Petersen RM, Canale DM, et al (2005) Incidence of *Trypanosoma cruzi* infection among children following domestic reinfestation after insecticide spraying in rural northwestern Argentina. Am J Trop Med Hyg 73: 95–103. 16014842 PMC1351233

[59] Aiga H, Sasagawa E, Hashimoto K, Nakamura J, Zúniga C, et al (2012) Chagas disease: assessing the existence of a threshold for bug infestation rate. Am J Trop Med Hyg 86: 972–979. 22665603 10.4269/ajtmh.2012.11-0652 PMC3366542

[60] Organización Panamericana de la Salud (2006) XVa Reunión de la Comisión Intergubernamental del Cono Sur para la Eliminación de *Triatoma infestans* y la Interrupción de la Transmisión de Tripanosomiasis Transfusional (INCOSUR-Chagas) (Brasilia, Brasil, 6–9 junio 2006). Conclusiones, Recomendaciones y Decisiones. Montevideo: Organización Panamericana de la Salud.

[61] Davis P, Howden-Chapman F (1996) Translating research findings into health policy. Soc Sci Med 43: 865–872. 8870150 10.1016/0277-9536(96)00130-x

[62] Cash DW, Clark WC, Alcock F, Dickson NM, Eckley N, et al (2003) Knowledge systems for sustainable development. Proc Natl Acad Sci USA 100: 8086–80891. 12777623 10.1073/pnas.1231332100 PMC166186

[63] Doyal L (1979) The Political Economy of Health. London: Pluto Press.

