# Supplementary material for: Intensified Surveillance and Insecticide-based Control of the Chagas Disease Vector Triatoma infestans in the Argentinean Chaco
Source: PLoS Negl Trop Dis. 2013 Apr 11;7(4):e2158. doi: 10.1371/journal.pntd.0002158 (PMC3623707; doi:10.1371/journal.pntd.0002158)
Supplement: Figure S1 — Examples to illustrate persistent infestations with T. infestans at house-compound level. Each row corresponds to a georeferenced (identifiable) site. Only sites ever found infested at least once in the selected houses are shown. Colors indicate infestation status at each house and survey according to collection method. (DOC) [file pntd.0002158.s001.doc]

**Figure S1. Examples to illustrate persistent infestations with *T. infestans* at house-compound level.** Each row corresponds to an identified, georeferenced site. Only sites ever found infested in the selected houses are shown. Colors indicate infestation status of each site at each survey according to collection method as displayed in legend.

|  |  | Months post-spraying | | | | | | | |  |  |  |
| --- | --- | --- | --- | --- | --- | --- | --- | --- | --- | --- | --- | --- |
| House | Site/ecotope | 0 | 4 | 8 | 12 | 17 | 22 | 28 | 35 |  |  |  |
| A | Kitchen | 5/3 | 0/1 |  |  |  |  |  |  |  |  |  |
|  | Domicile-1 | 4/2 | 1/1 | 0/5 |  |  |  |  |  |  | Legend | |
|  | Domicile-2 |  |  |  | 4/0 |  |  |  |  |  |  | |
|  | Chicken coop |  | 2/8 | 4/3 |  | 19/0 |  |  |  |  |  | Timed-manual collections |
|  |  |  |  |  |  |  |  |  |  |  |  | Knock-down collections or during insecticide application |
| B | Latrine |  | 0/1 |  |  |  |  |  |  |  |  |
|  | Pig pen |  |  |  |  | 2/1 |  |  |  |  |  |
|  | Kitchen |  |  |  |  | 5/2 |  |  |  |  |  | Householders’ collections |
|  | Domicile | 0/12 |  |  |  | 19/4 |  |  |  |  |  |  |
|  | Chicken coop |  |  | 4/1 |  |  |  |  |  |  |  | No bugs collected |
|  | Piled material |  |  | 0/2 |  |  |  |  |  |  |  |  |
|  | ‘Nidero’ | 1/3 | 3/3 | 4/0 |  | 14/2 |  |  |  |  |  | Site sprayed |
|  | Granary |  |  |  | 4/2 | 0/1 |  |  |  |  |  |  |
|  |  |  |  |  |  |  |  |  |  |  | 5/3 | No. of nymphs/adults collected |
| C | Storeroom |  |  | 1/3 |  |  |  |  |  |  |  |  |
|  | Domicile |  |  | 0/1 |  |  |  |  |  |  |  |  |
|  | ‘Nidero’ |  |  | 0/1 |  | 11/4 | 2/0 |  |  |  |  |  |
