# Supplementary material for: Intensified Surveillance and Insecticide-based Control of the Chagas Disease Vector Triatoma infestans in the Argentinean Chaco
Source: PLoS Negl Trop Dis. 2013 Apr 11;7(4):e2158. doi: 10.1371/journal.pntd.0002158 (PMC3623707; doi:10.1371/journal.pntd.0002158)
Supplement: Table S1 — Number of houses inhabited, inspected by timed manual collections (TMC) and sprayed with insecticides in Pampa del Indio. (DOC) [file pntd.0002158.s002.doc]

**Table S1.** **Number of houses inhabited, inspected by timed manual collections (TMC) and sprayed with insecticides in Pampa del Indio.**

| Months post-spraying | Inhabited houses | | | No. of uninhabited houses |  |
| --- | --- | --- | --- | --- | --- |
|  | No.  censused | No. (%) inspected by TMC | No. sprayed |  |  |
| 0 | 329 | 329 (100) | 325 | 24^a^ |  |
| 4 | 325 | 319 (98) | 0 | 48 |  |
| 8 | 325 | 319 (98) | 49 | 60 |  |
| 12 | 324 | 304 (94) | 20 | 62 |  |
| 17 | 322 | 309 (96) | 27 | 68 |  |
| 22 | 323 | 313 (97) | 8 | 70 |  |
| 28 | 319 | 290 (91) | 11 | 74 |  |
| 35 | 319 | 290 (91) | 0 | 74 |  |

^a^ 23 of these houses were sprayed with pyrethroids. Of the 5 houses not sprayed at 0 MPS, 4 had been sprayed with insecticides recently by others and 1 was vacant.
