# Supplementary material for: Intensified Surveillance and Insecticide-based Control of the Chagas Disease Vector Triatoma infestans in the Argentinean Chaco
Source: PLoS Negl Trop Dis. 2013 Apr 11;7(4):e2158. doi: 10.1371/journal.pntd.0002158 (PMC3623707; doi:10.1371/journal.pntd.0002158)
Supplement: Table S2 — Insecticide use between successive surveys as reported by householders. (DOC) [file pntd.0002158.s003.doc]

**Table S2. Insecticide use between successive surveys as reported by householders.**

| Months postspraying | Date | % of households reporting insecticide use (No.) | |
| --- | --- | --- | --- |
|  |  | Yes | NK/NA |
| 4 | Apr/08 | 48.5 (133) | (51) |
| 8 | Aug/08 | 41.4 (122) | (30) |
| 12 | Dec/08 | 42.5 (126) | (27) |
| 17 | May/09 | 69.0 (212) | (15) |
| 22 | Oct/09 | 55.8 (171) | (17) |

“NK/NA”: does not know/does not answer. Percentages were calculated relative to the number of effective answers (i.e., Yes or No).
