# Supplementary material for: Intensified Surveillance and Insecticide-based Control of the Chagas Disease Vector Triatoma infestans in the Argentinean Chaco
Source: PLoS Negl Trop Dis. 2013 Apr 11;7(4):e2158. doi: 10.1371/journal.pntd.0002158 (PMC3623707; doi:10.1371/journal.pntd.0002158)
Supplement: Text S1 — Details on relevant cases regarding insecticide spraying effectiveness and householders' practices. (DOC) [file pntd.0002158.s004.doc]

**Text S1. Relevant study cases related to the effectiveness of insecticide spraying and householders’ behavior in relation to infestations.**

Some limitations of pyrethroid sprays in suppressing local infestations were apparent in the following cases:

i) Of six sites found infested after selective pyrethroid sprays conducted at 8 or 12 MPS (i.e., when only infested sites were treated), three also had persistent infestations after two or more pyrethroid sprays (a granary, a kitchen and a ‘nidero’ sprayed once with a double dose of pyrethroids); a domicile sprayed twice with a standard dose; a storeroom sprayed once with a standard dose (at 0 MPS) and once with a double dose (at 8 MPS), and an old mud house used as a chicken coop that was sprayed twice with a standard dose and twice with a double dose of pyrethroids (i.e., experimental site reported in [33]).

ii) Difficulties for performing insecticide applications adequately: the persistent infestation of a domicile infested at 0, 4 and 8 MPS was most probably related to the impossibility of spraying properly the adjacent granary, which was half-full with recently harvested corn. Another granary full of corn and sprayed at 0 MPS was found infested at 4 MPS.

Other difficulties were registered with malathion selective sprays:

iii) A latrine and a ‘nidero’ sprayed with malathion at 22 MPS that subsequently had a persistent infestation. A sudden rainstorm fell the same night these two sites were sprayed. Both of them had deficient roofs and signs of water running on the surface of mud walls.

There were also some relevant cases associated with householders’ behavior in response to the recurrent presence of *T. infestans*, acknowledged by them as the main cause for these changes:

iv) Spontaneous actions undertaken by householders were associated with the suppression of specific foci at nine houses, several of which had had persistent infestations. These included five cases related to habitat (i.e., site) removal or modification (including the two sites persistently infested after the first malathion application: the ‘nidero’ was subsequently burnt, and the latrine’s roof was repaired to prevent the rain from washing out insecticide deposits on the walls); three cases related to change or removal of hosts or minimizing the time they spent in the site (e.g., recently born fledglings previously kept in the veranda were kept there only for a few days until they were able to stay in the chicken coop), and one case related to changes in host management practices and site construction.

v) One house with a persistent infestation at 4 MPS was vacated before the selective spray of the infested domicile at 8 MPS; no infestation was found in this house in a subsequent survey, although the nearest house at ~60 m had a persistent infestation.
